# Roles of a CCR4–NOT complex component GmNOT4-1 in regulating soybean nodulation

**DOI:** 10.3389/fpls.2023.1172354

**Published:** 2023-06-05

**Authors:** Jiangtao Zheng, Lili Sun, Dongmei Wang, Lin He, Weijun Du, Shujin Guo, Lixiang Wang

**Affiliations:** ^1^ College of Agronomy, Shanxi Agricultural University, Taigu, China; ^2^ State Key Laboratory of Crop Stress Adaptation Improvement, School of Life Sciences, Henan University, Kaifeng, China

**Keywords:** legume symbiotic nitrogen fixation, Ccr4-not complex, root nodule (symbiotic), CRISPR/Cas9, nod factor signalling

## Abstract

Legume-rhizobial symbiotic nitrogen fixation is the most efficient nitrogen assimilation system in the ecosystem. In the special interaction between organ–root nodules, legumes supply rhizobial carbohydrates for their proliferation, while rhizobials provide host plants with absorbable nitrogen. Nodule initiation and formation require a complex molecular dialogue between legumes and rhizobia, which involves the accurate regulation of a series of legume genes. The CCR4–NOT complex is a conserved multi-subunit complex with functions regulating gene expression in many cellular processes. However, the functions of the CCR4–NOT complex in rhizobia–host interactions remain unclear. In this study, we identified seven members of the *NOT4* family in soybean and further classified them into three subgroups. Bioinformatic analysis showed that *NOT4s* shared relatively conserved motifs and gene structures in each subgroup, while there were significant differences between *NOT4s* in the different subgroups. Expression profile analysis indicated that *NOT4s* may be involved in nodulation in soybean, as most of them were induced by Rhizobium infection and highly expressed in nodules. We further selected *GmNOT4-1* to clarify the biological function of these genes in soybean nodulation. Interestingly, we found that either *GmNOT4-1* overexpression or down-regulation of *GmNOT4-1* by RNAi or CRISPR/Cas9 gene editing would suppress the number of nodules in soybean. Intriguingly, alterations in the expression of *GmNOT4-1* repressed the expression of genes in the Nod factor signaling pathway. This research provides new insight into the function of the *CCR4–NOT* family in legumes and reveals *GmNOT4-1* to be a potent gene for regulating symbiotic nodulation.

## Background

Nitrogen is one of the essential macroelements for plant growth and development (Jia et al., 2017). Therefore, improving nitrogen utilization efficiency and appropriately applying nitrogen fertilizer are important guarantees for a high and stable yield of crops (Chun et al., 2005). In the ecosystem, given that leguminous plants have a high demand for nitrogen, they evolved an additional special root organ–root nodules—to fix atmospheric nitrogen and provide nitrogen to improve legume development (Ren et al., 2019).

Nodulation is a complex biological process involving direct interactions between rhizobial and legume signals (Howard, 1991). Nodulation is initiated by the flavonoids secreted by the legume roots, which were perceived by the surrounding compatible rhizobium strains and stimulated them to synthesize lipochitin oligosaccharides, called Nod factors (NF, Patra et al., 2017), which were sensed by Nod factor receptors (NFRs) (e.g., NF Perception [NFP] in *M. truncatula*, NF Receptors 1 and 5 [NFR1/5] in *L. japonicus*, and NFR1/5α in soybean) located on legume root hairs. This interaction then stimulated the consequent NF signaling pathway, which promoted root hair deformation, infection thread formation, outer cortical cell division, and root nodule primordia formation (Gao et al., 2002; Chou and Wei, 2010). Nodulation is a high-energy-consumption biological and host-dominant process; thus, legumes evolved an auto-regulation of nodulation (AON) mechanism; in brief, when the root nodules reach a certain number, the host will generate the CLE peptides as a signal molecular (Carroll et al., 2016), which would transmit to the legume shoot and be perceived by the NARK receptor. Furthermore, this recognition will generate shoot-derived molecular signals (e.g., cytokinin, miRNA2111), which transmit back to the roots and attenuate the nodulation process (Chou and Wei, 2010; Yuan et al., 2016).

Nodules are produced via a complex genetic program to allow rhizobial recognition and nodule formation, a series of transcription factors modulate the downstream responses to NF signaling, including NIN (L. Yuan et al., 2016; Wang et al., 2019), IPD3 (Interacting Protein of DMI3; Horváth, 2011), ERN1/ERN2/ERN3 (ERFs Required for Nodulation; Andriankaja et al., 2007; Middleton et al., 2007), NF-YA1 (Nuclear Factor-Y Subunit A1; Laloum et al., 2013), the NSP1 (Nodulation Signaling Pathway1) and NSP2 (Kaló et al., 2005; Smit et al., 2005; Oldroyd & Downie, 2008; Heckmann et al., 2011), NNC1 (Nodule number control1), and the DELLAs (Fonouni-Farde et al., 2016; Jin et al., 2016).

The ubiquitin–proteasome system is the most efficient and specific protein degradation mode for regulating plant growth and development. *ASTRAY and SINAT5* encoding RING-finger domains containing E3 ubiquitin ligase have been shown to function during legume nodulation (Fujita and Kawaguchi, 2002; Nishimura et al., 2002). The no-nodule alfalfa mutants *rh2* and *LjnsRING*, encoding RING-H2 domain-containing proteins (Shimomura et al., 2006), and LIN (in Medicago) and CERBERUS (in Lotus) shared 86% homology and both contained a U-box domain (Kiss et al., 2010). Plant U-box protein1 (PUB1) has the activity of an E3 ligase and interacts with LYK3/NFR1/DMI2/SYMRK to inhibit the infection of rhizobia and mycorrhizal fungi through its ubiquitination activity (Vernié et al., 2016).

The carbon catabolite repression 4–negative on TATA-less (CCR4–NOT) complex multi-subunit complex functions as a major regulator of gene expression homeostasis through ubiquitination (CCR4 and CAF1 [CCR4 associated factor 1]) and deadenylation (NOT4) in eukaryotes. In plants, core components of the CCR4–NOT complex were identified and revealed that Arabidopsis possesses AtCCR4–NOT complexes involved in mRNA recognition, AtCCR4-CAF1 has mRNA deadenylase activity to regulate environmental stresses (Liang et al., 2008; Walley et al., 2010; Suzuki et al., 2015), and NOT9B and CCR4–NOT can respond to far-red light and are involved in phyA-modulated gene expression. However, the identification of NOT4 in the complex and its function in legumes are yet to be unveiled.

In this study, genome-wide systematic characterization, including protein properties, chromosome distribution, phylogenetic relationship, protein motif, and gene expression pattern, was performed to study the soybean CCR4–NOT complex gene family. We selected *GmNOT4-1*, a member of the CCR4–NOT complex gene family that is highly expressed in nodules and significantly stimulated by Rhizobium infection, to determine its function in symbiotic nodulation using overexpression, RNAi, and CRISPR/Cas9. We found that in transgenic hairy roots harboring *GmNOT4-1*-overexpressing or in roots carrying *GmNOT4-1*-RNAi and CRISPR/Cas9-*GmNOT4-1*, the number of root nodules was significantly inhibited, and the marker genes for both the NF and AON signaling pathways were repressed. In conclusion, this study reports for the first time the function of the key eukaryotic gene expression regulatory complex CCR4–NOT in legume nodulation and identifies a distinguished nodulation regulator, *NOT4-1*, whose expression balance is relevant to soybean symbiosis.

## Materials and methods

### Plant materials and growth, hairy root transformation, and inoculation of soybean rhizobium

In this study, soybean [*G. max* (L.) *Merrill* cv. Williams 82] and *Agrobacterium rhizogenes* strain K599 were used for the hairy root transformation. The hairy root transformation procedure was previously described (Wang et al., 2014) with some modifications. The positive transformed composite plants were cultured in a low-nitrogen nutrient solution for 5 days for recovery; after that, the plants were transferred to vermiculite for inoculation with a suspension of *B. japonicum* strain USDA110 (30 ml, OD600 = 0.08). Nodule numbers were evaluated at 28 DAI (days after inoculation).

### Identification of *NOT4* gene family members in soybean

Genome data, protein sequence, and genome annotation files of *Glycine max* were downloaded from the Phytozome database (https://phytozome-next.jgi.doe.gov/). Protein families in the PANTHER database (http://www.pantherdb.org) were applied to download the Hidden Markov model (HMM) of the NOT transcription complex related family (PTHR12603). The soybean genome was searched using hmm search in HMMER (http://hmmer.org/) to identify NOT4 candidate genes (the screening threshold was 1.0e−10). The resulting candidate sequences were submitted to Intel Pro Scan (https://www.ebi.ac.uk/interpro/search/sequence-search) to check the PTHR12603 structure domain. Soybean *GmNOT4* family members were named according to their location distribution on soybean chromosomes.

### Phylogenetic analysis and chromosome mapping of *GmNOT4s in* soybean

NOT4 protein sequences from *Medicago truncatula*, *Phaseolus vulgaris*, *Arabidopsis thaliana*, soybean [*G. max* (L.)], and rice (*Oryza sativa*) NOT4 were downloaded from the Phytozome database (https://phytozome-next.jgi.doe.gov/). The phylogenetic tree of the *GmNOT4* gene family members of the five species was constructed by MEGA-X software and calculated by the NJ (neighbor-joining) method. The parameters were set to self-expanding and repeated 1,000 times. Chromosome distribution was analyzed using TBtools.

### Conserved motif and gene structure analysis

The MEME Suite online software (https://meme-suite.org/meme/) was used for conserved motif analysis, and the parameter was set to 10. The gene structure information of *GmNOT4s* was extracted from the soybean gene information GFF file, and the gene structures were visualized by TBtools.

### Gene expression

RNAprep Plant Plus Trizol Kit was used to extract RNA from collected samples, and the first-strand cDNA was synthesized using the Super Mix Kit (Hifair II 1 strand cDNA Synthesis SuperMix, gDNA digester plus) (Yeasen Biotech Co. Ltd., Shanghai, China). qPCR was performed using SYBR Green JumpStart Taq ReadyMix (Sigma-Aldrich). *GmCYP2* was used as an internal control (Jian et al., 2008). The primers used in this study are shown in **Table S1**.

### Plasmid construction

For the *GmNOT4-1* overexpression construct, the *GmNOT4-1* CDS fragment was inserted into the pCAMBIA1300-GFP vector through seamless cloning using the *Bam*H1 restriction site; for the *GmNOT4-1-*RNAi construct, the *GmNOT4-1* CDS fragment was ligated into the pDONOR207 entry vector, and then the target sequence was cloned into the pK7GWIW-GFP vector through the gateway LR reaction. For the *GmNOT4-1* CRISPR/Cas9 knock-out construct, *sgRNAs* were designed using *the* software Crispr-P (http://cbi.hzau.edu.cn/crispr/), and the top two reliable sgRNAs, CAAGGTGCGGTGAAGAGCA and TCGTCCTCTTCGCCTCTGC, were selected. Then, vector pCBC-DT1T2 was used as a template to clone the two CRISPR fragments, and the two obtained products were inserted into vector pKSE401-GFP.

### Statistical analysis

GraphPad Prism 7 (GraphPad Software, La Jolla, CA, USA) was used to analyze the data in this study. A Student’s *t*-test was performed to generate *P*-values. The statistical differences are marked as follows: *, *P <*0.05; **, *P <*0.01; ***, *P <*0.001.

## Results and discussion

### Identification and chromosomal distribution of soybean *NOT4* gene family

**Figure 1:**
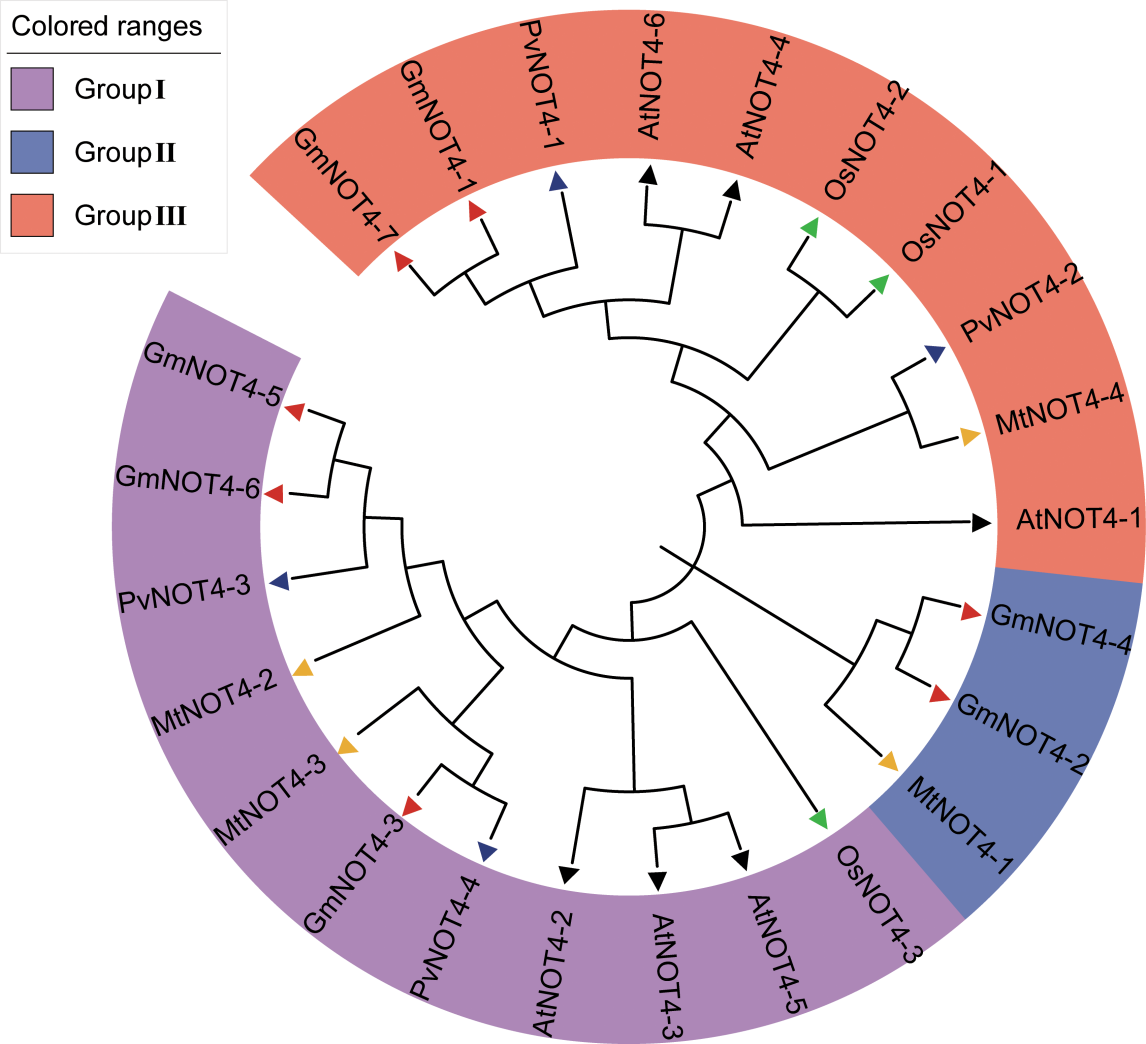
Phylogenetic tree of *NOT4* family members. NOT4 Protein sequences of *Medicago truncatula (Mt)*, *Phaseolus vulgaris (Pv)*, Arabidopsis thaliana (At), *Glycine max (Gm)* and *Oryza sativa (Os)* were divided into Group I to Group III and colored in purple, blue, and orange, respectively. Protein sequences were clustered using CLASTALW in MEGA 11.0. The phylogenetic tree was constructed by MEGA 11.0 using a bootstrap neighbor join method.

Seven *GmNOT4s* were identified from the soybean genome using BLAST and PANTHER searches and named *GmNOT4-1*–*GmNOT4-7* according to their positions on six chromosomes (**Figure S1**). The chromosomal distribution of *NOT4s* indicated that they were dispersed on chromosomes 5, 10, 12, 15, and 17. Except for *GmNOT4-4* and *GmNOT4-5*, which are on chromosome 13, each of the other five chromosomes contains one family member. The physical and chemical properties of *GmNOT4s* were analyzed, and it was found that the amino acid residues encoded by seven *GmNOT4s* ranged from 232 (GmNOT4-1) to 1,046 (GmNOT4-6), and the corresponding molecular weight ranged from 35,386.84 (GmNOT4-1) to 115,070.36 (GmNOT4-6) Da. The theoretical isoelectric points of seven *GmNOT4* family members ranged from 4.76 (GmNOT4-7) to 6.40 (GmNOT4-4), all of which belonged to weakly acidic proteins (**Table S2**). The total mean hydrophobic index was less than 0, indicating that they were hydrophilic proteins. To understand the functional characteristics and evolutionary relationship of the *NOT4* gene family, NOT4 protein sequences in soybean, *M. truncatula*, common bean, *A. thaliana*, and rice were retried to construct the phylogenetic tree. The results showed that the members of the *NOT4* gene family were divided into three subgroups (Groups I–III), in which groups I and III contained 10/11 members, and group II had three members. In the same subgroups, NOT4 proteins cluster together within species (**Figure 1**). Soybean *NOT4* family members were evenly distributed in three groups, with three members in Group I, two members in Group II, and two members in Group III.

### Conserved motifs and gene structure analysis of *NOT4* family genes in soybean

**Figure 2:**
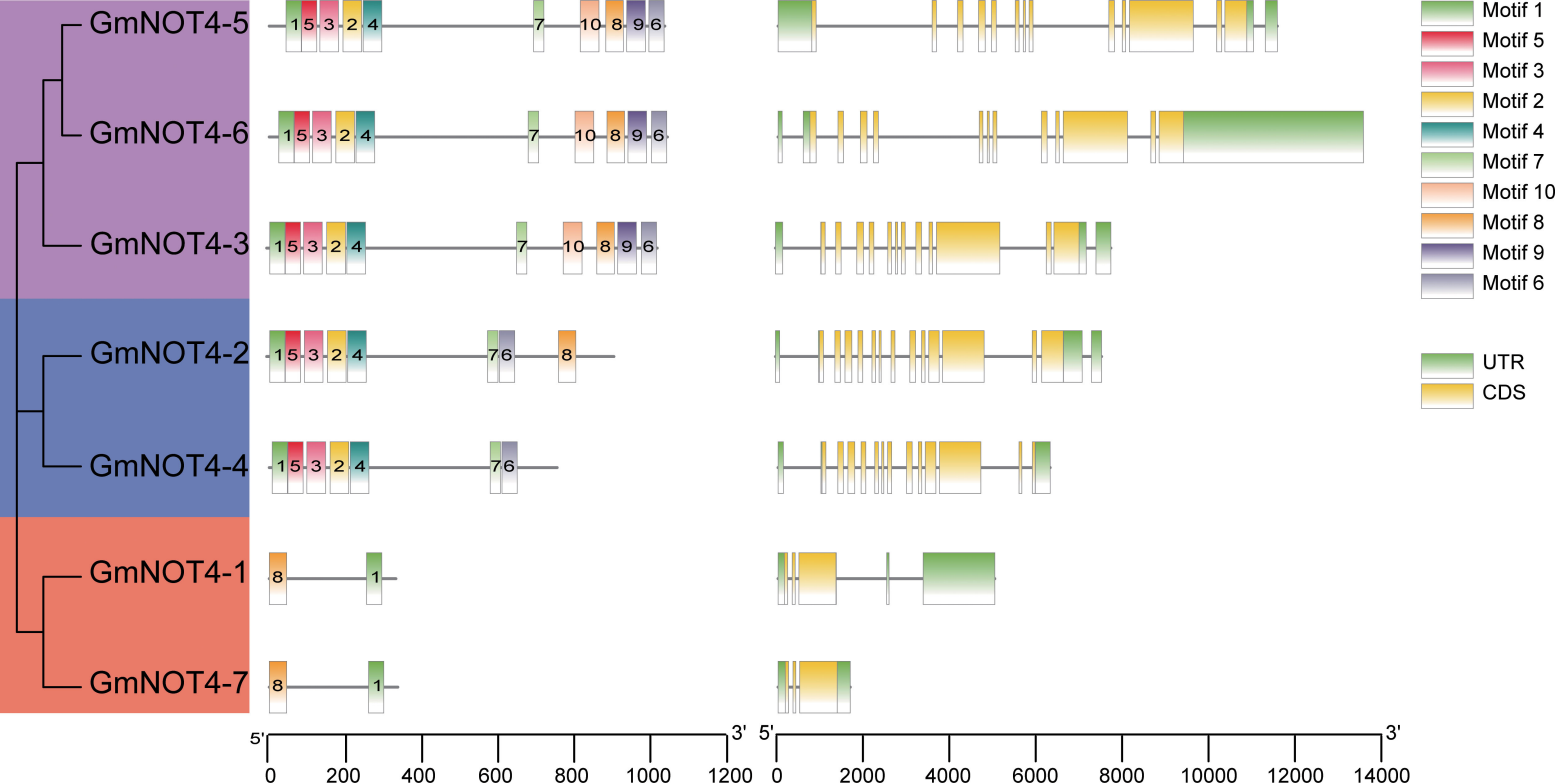
Gene structure and conserved motif distribution of *GmNOT4s*. MEME online software was used to identify conserved motifs. A total of 10 motifs were found in *GmNOT4s* genes and represented by different colored boxes and corresponding motif number, conserved amino acid sequence themes were shown below. (The left panel) shown the phylogenetic tree of group I–III genes in purple, blue, and orange colors; (The middle panel) shown the conserved motifs distribution in GmNOT4s; (The right panel) shown the gene structure of untranslated regions (UTRs) of the *GmNOT4s* gene are shown as green, yellow boxes and black lines.

To get a hint about the function of NOT4s, we first performed conserved motif analysis and gene structure analysis. There are 10 conserved motifs obtained from the GmNOT4 protein, and the number of motifs of GmNOT4s in different subgroups varied significantly, ranging from 2 to 10. Three members of Group I contain 10 motifs; there were eight motifs in GmNOT4-2 and seven for GmNOT4-4 in Group II and the members of Group III contain only two motifs. Among the 10 motifs, Motif1 was the most conserved one and was present in all GmNOT4 proteins (**Figure 2**). The variation in the types and amounts of conserved motifs in GmNOT4s reflects the functional diversity of these proteins. In addition, we have confirmed the conservation of motifs in NOT4 proteins in other plant species; we found these motifs are consistently present among Arabidopsis, rice, Medicago, and the common bean (**Figure S2**).

Diagram of *GmNOT4s* gene structures shows the exon number variation of GmNOT4s ranged from 3 to 13 (**Figure 2**). Combined with phylogenetic tree analysis, we found that genes with close genetic relationships mostly had similar gene structures. For example, all Group I GmNOT4s contained 12 exons, while Group III GmNOT4s contained three exons.

### *Cis*-acting element distribution in *GmNOT4s* promoters

Most transcriptional factors function in gene expression regulation by binding specific *cis*-acting elements in gene promoters to modulate gene expression. To predict the functions of GmNOT4 genes, 2-kilobase pairs upstream of the translational initiation site were selected as the promoter sequence and submitted to PlantCARE online software to predict the *cis*-acting elements (**Figure S3**). A total of 63 *cis*-acting elements were identified in seven *GmNOT4* promoters, while 54 *cis*-acting elements were related to plant hormones, stress, growth, and development. Firstly, core *cis*-acting elements exist in almost all promoters and consist of AT-TATA-box, CAAT-box, TATA-box, and TATA. The second group contains 26 cis-acting elements related to plant growth and development; all *GmNOT4* promoters contain photoreactive elements, which coincide with the CCR4–NOT complex response to far-red light. However, only *GmNOT4-1* and *GmNot4-7* contain *cis*-regulatory elements for flavonoid biosynthesis, which play a vital role in rhizobia attraction and symbiosis construction. Plant hormones are important for legume nodulation; 13 kinds of plant hormone related *cis*-acting elements were harbored in *GmNOT4* promoters; all *GmNOT4* promoters contained abscisic acid and ethylene-related *cis*-acting elements; and auxin response elements (TGA-elements), gibberellin response elements (P-box, GARE-motif) existed in most *GmNOT4s* promoters. Low oxygen conditions were very important for nitrogen fixation in nodules, and we found that among the stress response *cis* elements, *GmNOT4s* harbored *the* most anaerobic induction and stress-related *cis* elements. The specific *cis*-acting element distributions in *GmNOT4* promoters suggest their different biological functions.

### Gene expression pattern of *GmNOT4* genes

To investigate the potential function of *NOT4s* during soybean nodulation, we examined the temporal and spatial expression patterns of *GmNOT4s* by qRT-PCR.

**Figure 3:**
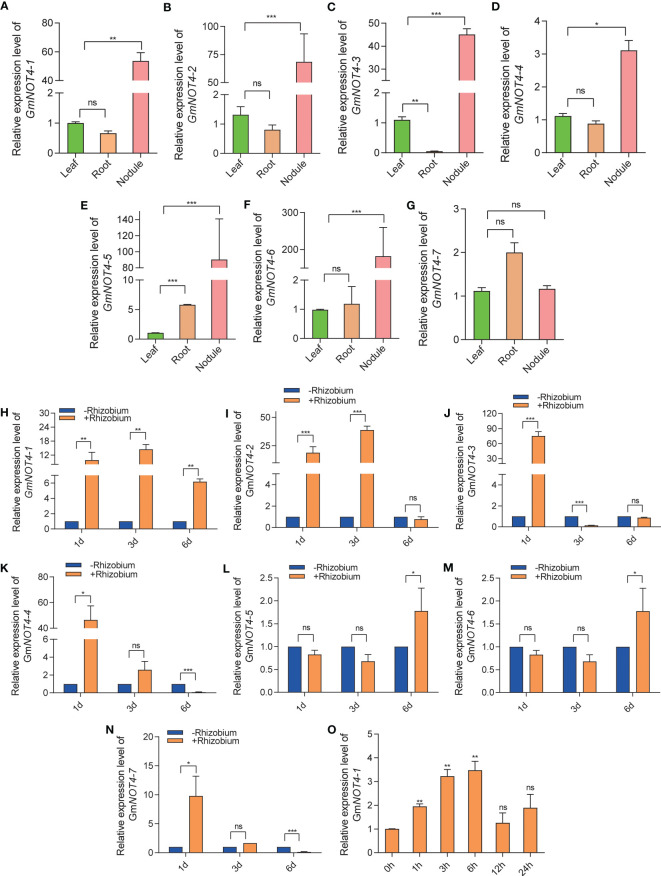
Expression pattern of soybean *GmNOT4s*. **(A–G)** show the relative expression level of *GmNOT4-1*, *GmNOT4-2*, *GmNOT4-3*, *GmNOT4-4*, *GmNOT4-5*, *GmNOT4-6*, *and GmNOT4-7* in soybean leaf, root, and nodule at 28 DAI, respectively. **(H–N)** show the relative expression level of *GmNOT4-1*, *GmNOT4-2*, *GmNOT4-3*, *GmNOT4-4*, *GmNOT4-5*, *GmNOT4-6*, *and GmNOT4-7* at 1, 3, and 6 DAI were validated by qPCR. **(O)** expression level of *GmNOT4-1* in soybean roots at 0, 1, 3, 6, 12, and 24 HAI (hours after inoculation). *GmCYP2* was used as an internal control. **(A–O)**, n = 12, Student’s t-test; *P <0.05; **P <0.01; ***P <0.001, ns, no significance).

We found that all but NOT4-7 were highly expressed in root nodules (**Figures 3A–F**). Further study has shown that the expression of *GmNOT4-1*, *GmNOT4-2*, *GmNOT4-3*, *GmNOT4-4*, *and GmNOT4-7* was dramatically stimulated by rhizobium infection at 1 DAI (**Figures 3H–K, N**). The expression of *GmNOT4-1 and GmNOT4-2* was induced by rhizobium USDA110 while *GmNOT4-3* was repressed at 3 DAI (**Figure 3H–J**). The expression of *GmNOT4-1*, *GmNOT4-5*, *and GmNOT4-6* was induced by rhizobium, while *GmNOT4-4* and *GmNOT4-7* were repressed at 6 DAI (**Figures 3H, L, M**). These results show that all *GmNOT4s* responded to rhizobium at least at one point. Combining the above tissue expression pattern to prove the function of *GmNOT4s in* legume root nodule symbiosis, we selected *GmNOT4-1 for* further study, whose expression was induced at all checkpoints within a short period of inoculation (1, 3, and 6 DAI).

### *GmNOT4-1* is an important regulator in regulating soybean nodulation

**Figure 4:**
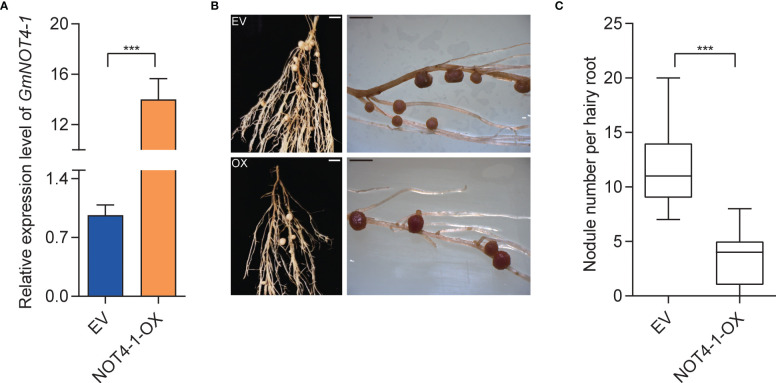
Phenotypic analysis of *GmNOT4-1* overexpression. **(A)** Expression level of transgenic hairy roots harboring empty vector and *35S:GmNOT4-1*. The expression levels were normalized against the housekeeping gene of soybean *GmCYP2*. Student’s *t*-test was performed (***p <0.001, n = 15). **(B)** Nodule status of individual transgenic roots expressing EV1 and *35S:GmNOT4-1* at 28 DAI. Bar = 2 mm. **(C)**, Quantitative analysis of nodule number per hairy root carrying EV and *35S: GmNOT4-1* at 28 DAI. Values are the mean ± SD. A total of 20 hairy roots were collected for each biological replicate (n = 12, Student’s *t*-test; ***p <0.001).

To genetically explore whether *GmNOT4-1* is involved in the regulation of soybean nodulation, overexpression and knockout/knockdown analyses of *GmNOT4-1* were performed using the hairy root transformation system. Firstly, we constructed *35S:GmNOT4-1 and* obtained overexpressing *GmNOT4-1* (*GmNOT4-1*-OX) roots by qPCR analysis (**Figure 4A**). The effects of *GmNOT4-1* overexpression on the early and late stages of nodulation were evaluated at 1, 6, and 28 days after inoculation (DAI) (**Figure S4**). Firstly, the expression of rhizobial infection-related genes, including *GmRPG*, *GmNPL*, *GmVPY*, *GmCYCLOPS*, and *GmSCARN*, was validated. As shown in **Figure S5**, all rhizobial infection-related genes we checked in this study were significantly inhibited in *GmNOT4-1* overexpressing roots. Then, we found the number of root hairs showing deformation was markedly decreased in *GmNOT4-1*-OX hairy roots at 6 DAI (**Figure S6**). Finally, the nodule numbers were quantified at 28 DAI. The average nodule number per *GmNOT4-1* overexpressed root was about 3.6, while empty vector (EV) control roots produced an average of 11.8 nodules per root, with an approximately 69% reduction by *GmNOT4-1* overexpression. These data suggest that *GmNOT4-1* plays a negative role in regulating soybean nodulation (**Figures 4B, C**).

**Figure 5:**
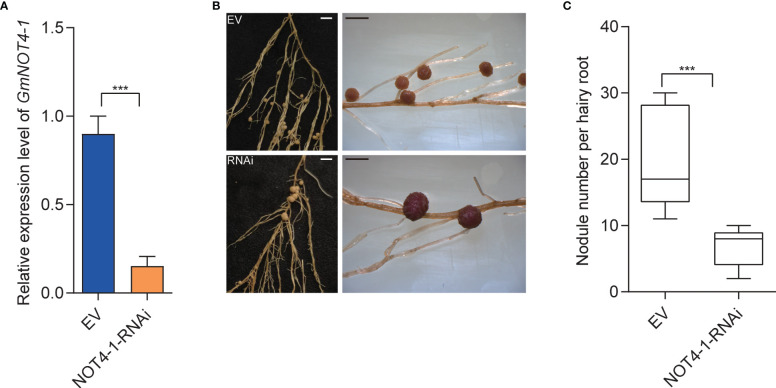
Knocking down *GmNOT4-1* inhibits nodulation. **(A)** qRT-PCR analysis of transgenic hairy roots harboring empty vector and *GmNOT4-1*-RNAi. The expression levels were normalized against the housekeeping gene of soybean *GmCYP2*. Student’s *t*-test was performed (***p <0.001, n = 15). **(B)** Nodule status of individual transgenic roots expressing empty vector and *GmNOT4-1*-RNAi at 28 DAI. Bar = 2 mm. **(C)** Quantitative analysis of nodule number per hairy root carrying empty vector and *GmNOT4-1*-RNAi at 28 DAI. Values are the mean ± SD. A total of 20 hairy roots were collected for each biological replicate (n = 12, Student’s *t*-test; ***p <0.001).

To further validate the function of *GmNOT4-1* during nodulation, we constructed the *NOT4-1*-RNAi to analyze the soybean nodulation phenotype when the expression of *GmNOT4-1* was reduced (**Figure S4**). As shown in **Figures 5B, C**, transgenic roots harboring*GmNOT4-1-*RNAi produced fewer nodules (6.9 nodules per root) than empty vector (EV) control roots (19.5 nodules per root), so that the nodule number per *GmNOT4-1*-silenced root was reduced by approximately 64.6%. Further, the CRISPR/Cas9 system was also applied to knock out *GmNOT4-1* in soybean hairy roots (**Figures S8**, **S9**), and the gene editing was verified by sequencing (**Table S3**). The expression of rhizobial infection-related genes, including *GmRPG*, *GmNPL*, *GmVPY*, *GmCYCLOPS*, and *GmSCARN*, was significantly inhibited in *GmNOT4-1-*KO roots. The number of deformed root hairs was markedly decreased in *GmNOT4-1* edited knock-out hairy roots at 6 DAI compared to the vector control (**Figure S6**). Finally, we found that the *GmNOT4-1* edited roots produce functional nodules (**Figure S7**) with a significantly reduced nodule number, which showed the same result as the *GmNOT4-1-*RNAi roots (**Figures 5B, C**).

Combined with the overexpression results, it is suggested that homeostasis of the expression of *GmNOT4-1* is critical for the regulation of soybean nodulation.

### Marker genes in the NF pathway were affected by *GmNOT4-1*

**Figure 6:**
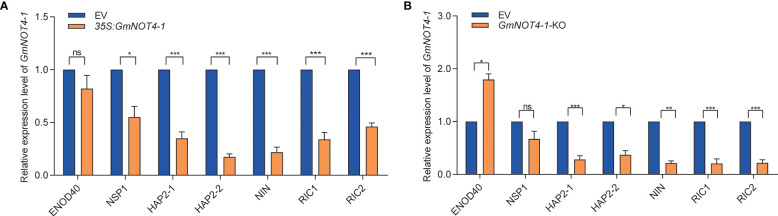
*GmNOT4-1* expression alliterating inhibit the transcript levels of nodulation-related genes. **(A)** qRT-PCR analysis of *ENOD40*, *GmNINa*, *NSP1*, *HAP2-1*, *HAP2-2*, *GmRIC1*, *and GmRIC2* in roots transformed with empty vector and *GmNOT4-1* at 6 DAI (n = 6). **(B)** qRT-PCR analysis of *ENOD40*, *GmNINa*, *NSP1*, *HAP2-1*, *HAP2-2*, *GmRIC1*, *and GmRIC2* in roots transformed with empty vector and *GmNOT4-1* knock out at 6 DAI (n = 6). We set transcript level of the *ENOD40*, *GmNINa*, *NSP1*, *HAP2-1*, *HAP2-2*, *GmRIC1*, *and GmRIC2* at 6 DAI EV hairy roots as “1.” The transcript amounts in each sample were normalized to those of *GmCYP2* (n = 6, Student’s t-test; *p <0.05, **p <0.01, and ***p <0.001; ns, no significance).

Nodule number was mainly modulated by both the NF signaling and AON signaling pathways. Given the phenotype that either overexpression or knockdown (out) of *GmNOT4-1* significantly decreased soybean nodule number, it is worth checking whether *GmNOT4-1* regulates nodulation through the above pathways. Thus, we examined the expression level of several nodulation marker genes in soybean, including NF signaling pathway genes *ENOD40*, *GmNINa*, *NSP1*, *HAP2-1*, and *HAP2-2*, and AON signaling pathway genes *GmRIC1/2*. In general, we found that either overexpression or silence of *GmNOT4-1* resulted in reduced expression of nodulation marker genes compared with the empty vector control roots (**Figure 6**).

Legumes plants can specifically interact with the phylogenetically diverse group of soil bacteria-rhizobia to form nodules. However, symbiotic nitrogen fixation is a highly energy-intensive biological process; thus, host legumes have evolved a root–shoot–root long-distance auto-regulation of nodulation (AON) system to refine the number of nodules (Patra et al., 2017; Ren et al., 2019). NODULE INCEPTION (NIN) induces the expression of CLE ROOT SIGNAL1 (*CLE-RS1*) and *CLE-RS2* to activate AON to inhibit excessive nodulation (Chun et al., 2005). NIN also modulates almost all nodulation processes, including nodule initiation, nodule organogenesis, and nitrogen fixation. Thus, NIN can act as a bifunctional transcription factor, fine-tuning legume nodulation. Interestingly, in this study, we observed that *GmNOT4-1* also function as a biofunctional regulator in nodulation, both overexpression and CRISPR/Cas9 knock out of *GmNOT4-1* inhibited the number of nodules in soybean. Except for *GmNOT4-1*, we found all *GmNOT4s* responded to rhizobium at least at one time point (**Figure 3**). We do not exclude the possibility that different family members might have a role in a particular stage of root nodule symbiosis.

In previous studies, a series of transcriptional factors and microRNAs were identified (NIN, NSP1/2, miR172c, etc.) that modulate the expression of some key nodulation regulators (*ERN1*, *ENOD40*, *RICs*, etc.) and finally legume nodule number. In eukaryotic cells, except for transcriptional factors and miRNAs, the multiprotein complex CCR4–NOT also plays a vital role in regulating gene expression *via the* shortening of poly(A) tails of messenger RNA. In this study, we identified *GmNOT4-1*, one component of the soybean CCR4–NOT complex, whose expression homeostasis is important for soybean nodule initiation and subsequent nodule number (**Figures 4**, **5**; **S4**-**S6**). We found that either overexpression or silencing of *GmNOT4-1* resulted in reduced expression of nodulation marker genes compared with empty vector control roots (**Figure 6**). *RIC1/2* was induced by nodule primordial formation to inhibit excessive nodulation; *GmNOT4-1* may function before nodule primordial formation; thus, the resulting *RIC1/2* induction and AON signaling were blocked by the overexpression or silencing of *GmNOT4-1*. Further study is needed to clarify the other components of the CCR4–NOT complex, including GmNOT4 family members in nodulation, and construct a direct link between the CCR4–NOT complex and nodulation signaling pathway genes. In addition, *GmNOT4-1* encodes a RING domain containing an E3 ligase; it may function by adjusting substrate protein levels. Identification of the target of GmNOT4-1 can further unveil its role in the process.

## Conclusions

In this research, we identified seven members of the *NOT4* family in soybeans and found that *GmNOT4-1* was mainly expressed in soybean nodules. Interestingly, we observed that both overexpression and downregulation of *GmNOT4-1* inhibited the number of nodules in soybean. The CRISPR/Cas9 system was applied to validate this phenotype. Finally, we demonstrated that alterations in *GmNOT4-1* expression level repressed the expression of genes in the Nod factor signaling pathway. To our best knowledge, this is the first research to study the CCR4–NOT complex in legume nodulation. Like the founder transcription factor *NIN* (NODULE INCEPTION), essential for nodulation, the gene expression level of *NIN* was rigidly regulated for different processes of nodulation, including rhizobial infection, nodule organogenesis, and AON signaling. *GmNOT4-1* showed the same phenotype pattern as the *NIN* gene; overexpression and knockdown (out) both inhibit the proper nodulation. The further detailed phenotype and mechanism study will provide a better understanding of *GmNOT4-1* and its roles in nodulation.

This research will provide novel insight into the function of the *CCR4–NOT* family in legumes and reveal *GmNOT4-1* to be a valuable gene in regulating symbiotic nodulation.

## Data availability statement

The original contributions presented in the study are included in the article/**Supplementary Material**. Further inquiries can be directed to the corresponding authors.

## Author contributions

WLX, GSJ, and DWJ designed and conceived the study. ZJT cloned the gene and constructed the vector, grew the seedlings, and harvested them for quantitative detection. SLL completed the bioinformatics analysis. WDM and HL participated in the expression analysis. All authors contributed to the article and approved the submitted version.

## Supplementary material

Supplementary Figure 1 Chromosomal distribution of *NOT4* gene family members *7 GmNOT4s* were identified from soybean genome and named *GmNOT4-1* to *GmNOT4-7* according to their distribution positions on chromosomes.

Supplementary Figure 2 Motif distribution of *GmNOT4s* NOT4 Protein sequences of Medicago truncatula (Mt), Phaseolus vulgaris (Pv), Arabidopsis thaliana (At), Glycine max (Gm) and Oryza sativa (Os) were subjected into MEME online software to identify conserved motifs. 10 motifs were found in NOT4s genes and represented by different colored boxes and corresponding motif number, conserved amino acid sequence themes were shown below. (The left panel) shown the phylogenetic tree of group I~III genes in purple, blue, and orange colors; (The middle panel) shown the conserved motifs distribution in NOT4s; (The right panel) shown the gene structure of untranslated regions (UTRs) of the NOT4s gene are shown as green, yellow boxes and black lines.

Supplementary Figure 3
*Cis*-acting elements of soybean *GmNOT4s* gene promoter Promoter sequences (2000 base pair upstream of start codon site) of *GmNOT4s* were submitted to PlantCARE database to identify *cis*-acting elements. Boxes with different colors indicate various *cis*-acting elements located in the promoter regions of *GmNOT4s*. Abbreviations: AT~TATA-box, CAAT-box, TATA-box, TATA: core *cis*-elements; Box 4, GT1-motif, ACE, G-box: optical response element; CAT-box, CCGTCC-box: associated with meristem expression; circadian: circadian rhythm control; GCN4-Motif: endosperm expression; HD-Zip1: cell differentiation in palisade tissue; AC-I: xylem synthesis; MBSI: flavonoid biosynthesis; TGA-element: Auxin responsive element; ABRE, AAGAA-motif, ABRE3a: abscisic acid reaction; TCA, TCA-element: salicylic acid responsive elements; P-box, GARE-motif: gibberellin response element; TGACG-Motif, CGTCA-motif: methyl jasmonate responsive elements; ERE: ethylene response element; ARE: anaerobic induction element; MYB, Myb-binding site, MYC, MBS: Drought induction elements; LTR: low temperature element; TC rich repeats, CCAAT-box, as-1, STRE: stress reaction elements; WRE3, WUN-motif, W box: traumatic stress elements; A-box, CARE, CCGTCC motif, AT-rich sequence: unknown function.

Supplementary Figure 4 No combined and off-target effects existed in GmNOT4-1 overexpression or knoct-out roots (A), qRT-PCR analysis of GmNOT4-2, GmNOT4-3, GmNOT4-4, GmNOT4-5, GmNOT4-6 and GmNOT4-7 in roots transformed with empty vector and GmNOT4-1 overexpression at 6 DAI (n = 6). (B), qRT-PCR analysis of GmNOT4-2, GmNOT4-3, GmNOT4-4, GmNOT4-5, GmNOT4-6 and GmNOT4-7 in roots transformed with empty vector and GmNOT4-1-KO at 6 DAI (n = 6). We set transcript level of the GmNOT4-2, GmNOT4-3, GmNOT4-4, GmNOT4-5, GmNOT4-6 and GmNOT4-7 at 6 DAI EV hairy roots as “1”. The transcript amounts in each sample were normalized to those of GmCYP2 (n = 12, Student’s t-test; “ns” =No significance).

Supplementary Figure 5 GmNOT4-1 regulate nodulation though affecting rhizobial infection (A), qRT-PCR analysis of GmNPL, GmRPG, GmVPY, GmCYCLOPS and GmSCARN in roots transformed with empty vector and GmNOT4-1 overexpression at 6 DAI (n = 6). (B), qRT-PCR analysis of GmNPL, GmRPG, GmVPY, GmCYCLOPS and GmSCARN in roots transformed with empty vector and GmNOT4-1-KO at 6 DAI (n = 6). The transcript amounts in each sample were normalized to those of GmCYP2 (n = 12, Student’s t-test; *p < 0.05, **p < 0.01, and ***p < 0.001).

Supplementary Figure 6 GmNOT4-1 overexpression and knock out decreases the number of deformed root hairs **(A)**, Expression level of transgenic hairy roots harboring empty vector and 35S:GmNOT4-1. The expression levels were normalized against the housekeeping gene of soybean GmCYP2. Student’s t-test was performed (***p < 0.001, n = 20). **(B)**, At 6 DAI, 2 cm root segments of hairy roots overexpressing GmNOT4-1 or expressing EV below the root-hypocotyl junction were cut and stained with 1% (w/v) methylene blue. Deformed root hairs were counted (n=20). (B) Root hair deformation in transgenic roots harboring EV and 35S:GmNOT4-1 vector. Bar=40 μm. **(C)**, Quantification of deformed root hairs in the transgenic lines (n=10 to 12). Values are averages ± SD from three independent experiments. Asterisks represent statistically significant differences. (n = 20, Student’s t-test; ***p < 0.001). **(D)**, Expression level of transgenic hairy roots harboring empty vector and GmNOT4-1-KO. The expression levels were normalized against the housekeeping gene of soybean GmCYP2. Student’s t-test was performed (***p < 0.001, n = 20). **(E)**, Root hair deformation in transgenic roots harboring EV and GmNOT4-1-KO. Bar=40 μm. **(F)**, Quantification of deformed root hairs in the transgenic root harboring EV and GmNOT4-1-KO (n=20). Values are averages ± SD from three independent experiments. Asterisks represent statistically significant differences. (n = 20, Student’s t-test; ***p < 0.001).

Supplementary Figure 7 Nodules of GmNOT4-1 overexpression and RNAi roots are functional Nodule performance of mature nodule in roots expressing empty vector, overexpression, and GmNOT4-1-KO at 28 DAI. Bar =2 mm.

Supplementary Figure 8 Phenotypic analysis of GmNOT4-1 gene editing **(A)**, Relative expression level of GmNOT4-1 in GmNOT4-1 edited roots; The expression levels were normalized against the housekeeping gene of soybean GmCYP2. Student’s t-test was performed (***p < 0.001, n = 15). **(B)**, Nodule performance of individual transgenic roots expressing empty vector and GmNOT4-1-KO at 28 DAI. Bar =2 mm. **(C)**, Quantitative data of nodule number per hairy root carrying empty vector and GmNOT4-1-KO at 28 DAI. Values are the mean ± SD. 20 hairy roots were collected for each biological replicate. (Student’s t-test; ***p < 0.001).

Supplementary Figure 9 Alignment of GmNOT4-1 gRNAs with other GmNOT4 family members **(A)**, The front gRNA sequence aligned with the corresponding region of other GmNOT4 family members. **(B)**, The back gRNA sequence aligned with the corresponding region of other GmNOT4 family members, the software Daneman was applied for the sequence alignment.
