# Supplementary figures and images for: Roles of a CCR4–NOT complex component GmNOT4-1 in regulating soybean nodulation
Source: Front Plant Sci. 2023 Jun 5;14:1172354. doi: 10.3389/fpls.2023.1172354 (PMC10277652; doi:10.3389/fpls.2023.1172354)

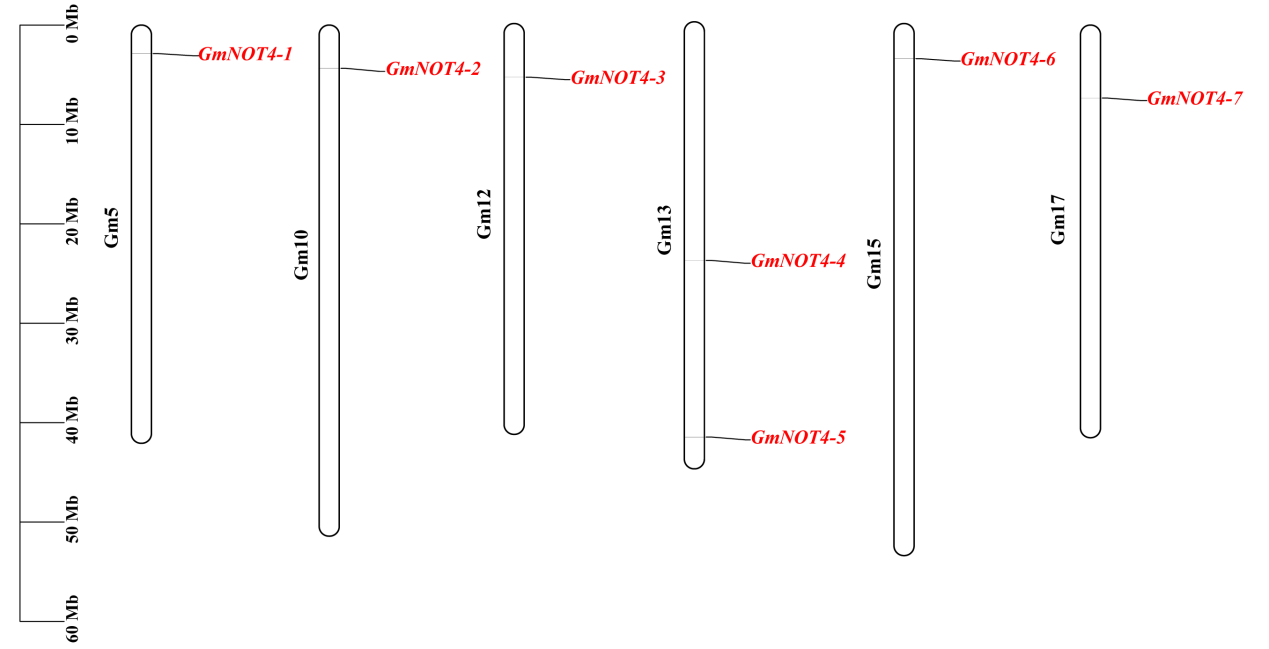

Supplement: Supplementary Figure 1 — Chromosomal distribution of NOT4 gene family members 7 GmNOT4s were identified from soybean genome and named GmNOT4-1 to GmNOT4-7 according to their distribution positions on chromosomes. [file Image_1.tif]

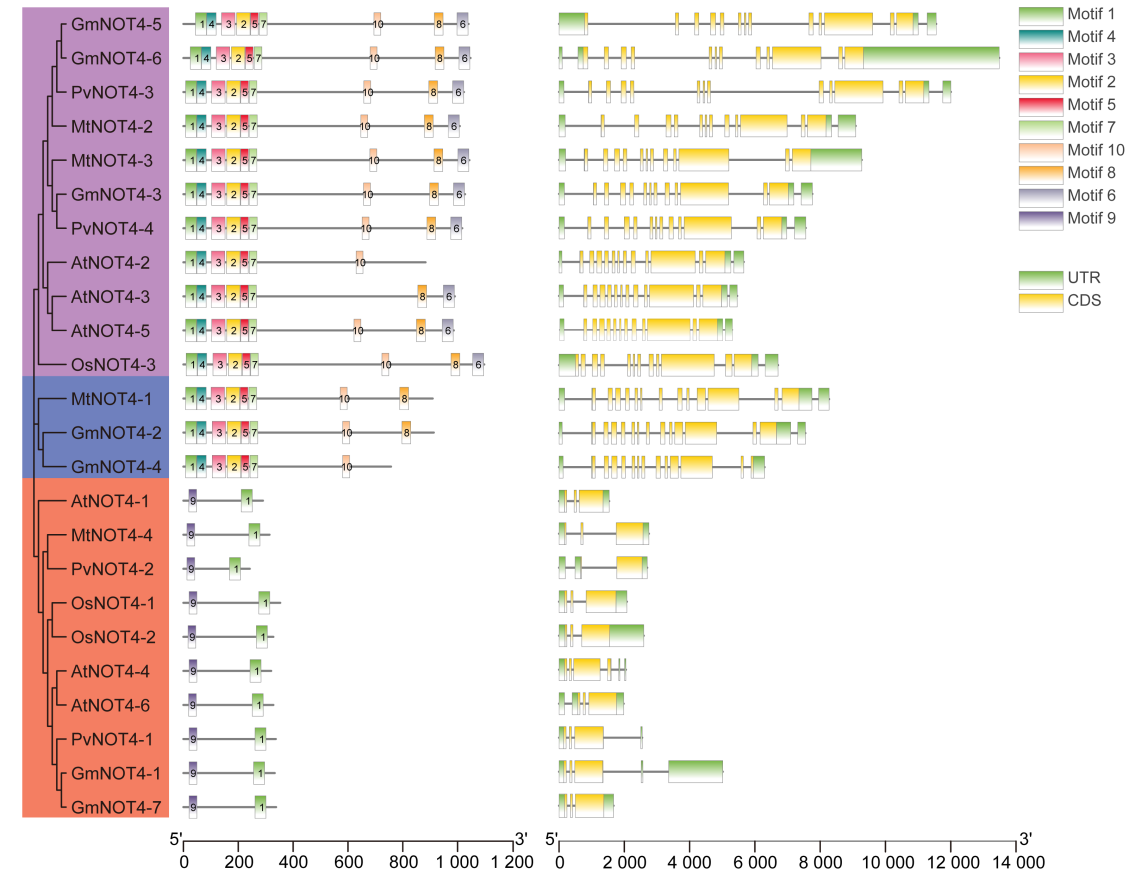

Supplement: Supplementary Figure 2 — Motif distribution of GmNOT4s NOT4 Protein sequences of Medicago truncatula (Mt), Phaseolus vulgaris (Pv), Arabidopsis thaliana (At), Glycine max (Gm) and Oryza sativa (Os) were subjected into MEME online software to identify conserved motifs. 10 motifs were found in NOT4s genes and represented by different colored boxes and corresponding motif number, conserved amino acid sequence themes were shown below. (The left panel) shown the phylogenetic tree of group I~III genes in purple, blue, and orange colors; (The middle panel) shown the conserved motifs distribution in NOT4s; (The right panel) shown the gene structure of untranslated regions (UTRs) of the NOT4s gene are shown as green, yellow boxes and black lines. [file Image_2.tif]

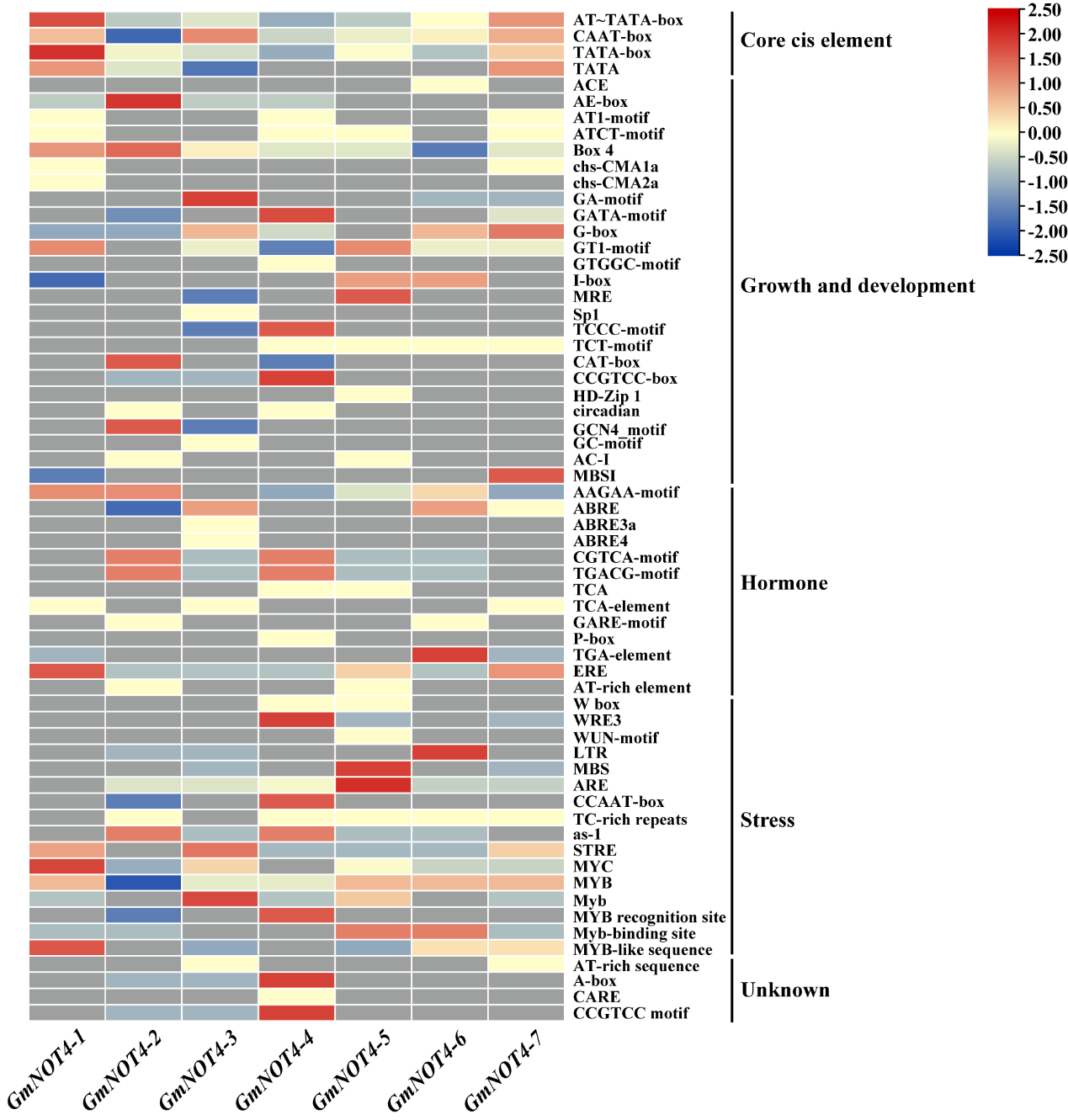

Supplement: Supplementary Figure 3 — Cis-acting elements of soybean GmNOT4s gene promoter Promoter sequences (2000 base pair upstream of start codon site) of GmNOT4s were submitted to PlantCARE database to identify cis-acting elements. Boxes with different colors indicate various cis-acting elements located in the promoter regions of GmNOT4s. Abbreviations: AT~TATA-box, CAAT-box, TATA-box, TATA: core cis-elements; Box 4, GT1-motif, ACE, G-box: optical response element; CAT-box, CCGTCC-box: associated with meristem expression; circadian: circadian rhythm control; GCN4-Motif: endosperm expression; HD-Zip1: cell differentiation in palisade tissue; AC-I: xylem synthesis; MBSI: flavonoid biosynthesis; TGA-element: Auxin responsive element; ABRE, AAGAA-motif, ABRE3a: abscisic acid reaction; TCA, TCA-element: salicylic acid responsive elements; P-box, GARE-motif: gibberellin response element; TGACG-Motif, CGTCA-motif: methyl jasmonate responsive elements; ERE: ethylene response element; ARE: anaerobic induction element; MYB, Myb-binding site, MYC, MBS: Drought induction elements; LTR: low temperature element; TC rich repeats, CCAAT-box, as-1, STRE: stress reaction elements; WRE3, WUN-motif, W box: traumatic stress elements; A-box, CARE, CCGTCC motif, AT-rich sequence: unknown function. [file Image_3.tif]

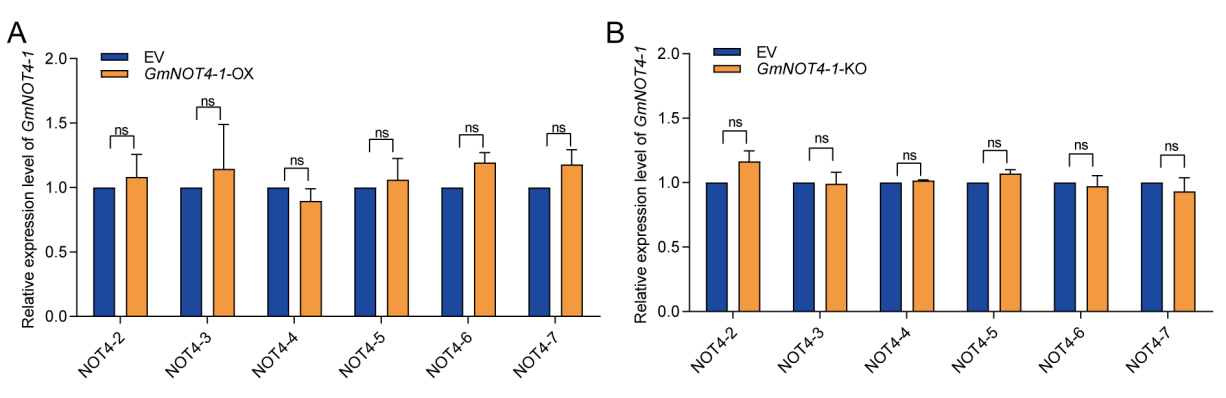

Supplement: Supplementary Figure 4 — No combined and off-target effects existed in GmNOT4-1 overexpression or knoct-out roots (A), qRT-PCR analysis of GmNOT4-2, GmNOT4-3, GmNOT4-4, GmNOT4-5, GmNOT4-6 and GmNOT4-7 in roots transformed with empty vector and GmNOT4-1 overexpression at 6 DAI (n = 6). (B), qRT-PCR analysis of GmNOT4-2, GmNOT4-3, GmNOT4-4, GmNOT4-5, GmNOT4-6 and GmNOT4-7 in roots transformed with empty vector and GmNOT4-1-KO at 6 DAI (n = 6). We set transcript level of the GmNOT4-2, GmNOT4-3, GmNOT4-4, GmNOT4-5, GmNOT4-6 and GmNOT4-7 at 6 DAI EV hairy roots as “1”. The transcript amounts in each sample were normalized to those of GmCYP2 (n = 12, Student’s t-test; “ns” =No significance). [file Image_4.tif]

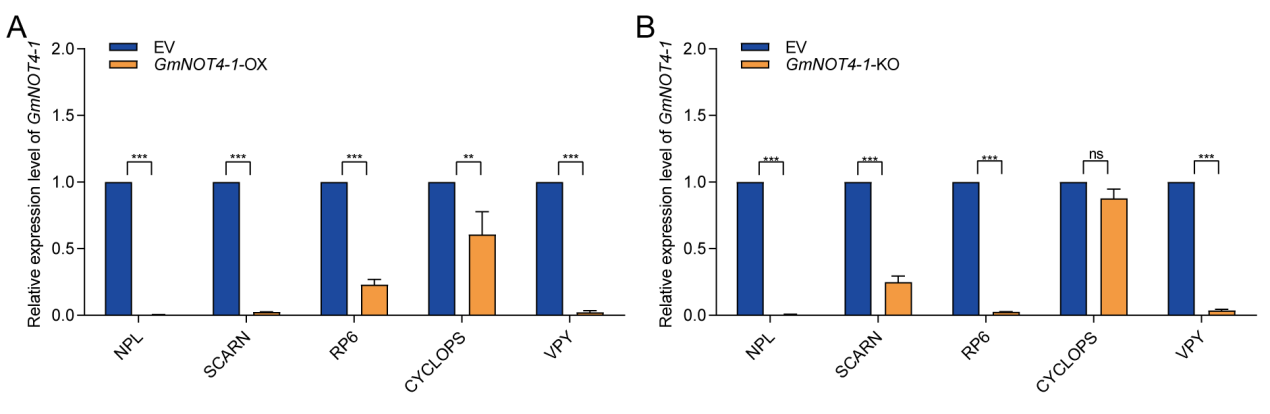

Supplement: Supplementary Figure 5 — GmNOT4-1 regulate nodulation though affecting rhizobial infection (A), qRT-PCR analysis of GmNPL, GmRPG, GmVPY, GmCYCLOPS and GmSCARN in roots transformed with empty vector and GmNOT4-1 overexpression at 6 DAI (n = 6). (B), qRT-PCR analysis of GmNPL, GmRPG, GmVPY, GmCYCLOPS and GmSCARN in roots transformed with empty vector and GmNOT4-1-KO at 6 DAI (n = 6). The transcript amounts in each sample were normalized to those of GmCYP2 (n = 12, Student’s t-test; *p < 0.05, **p < 0.01, and ***p < 0.001). [file Image_5.tif]

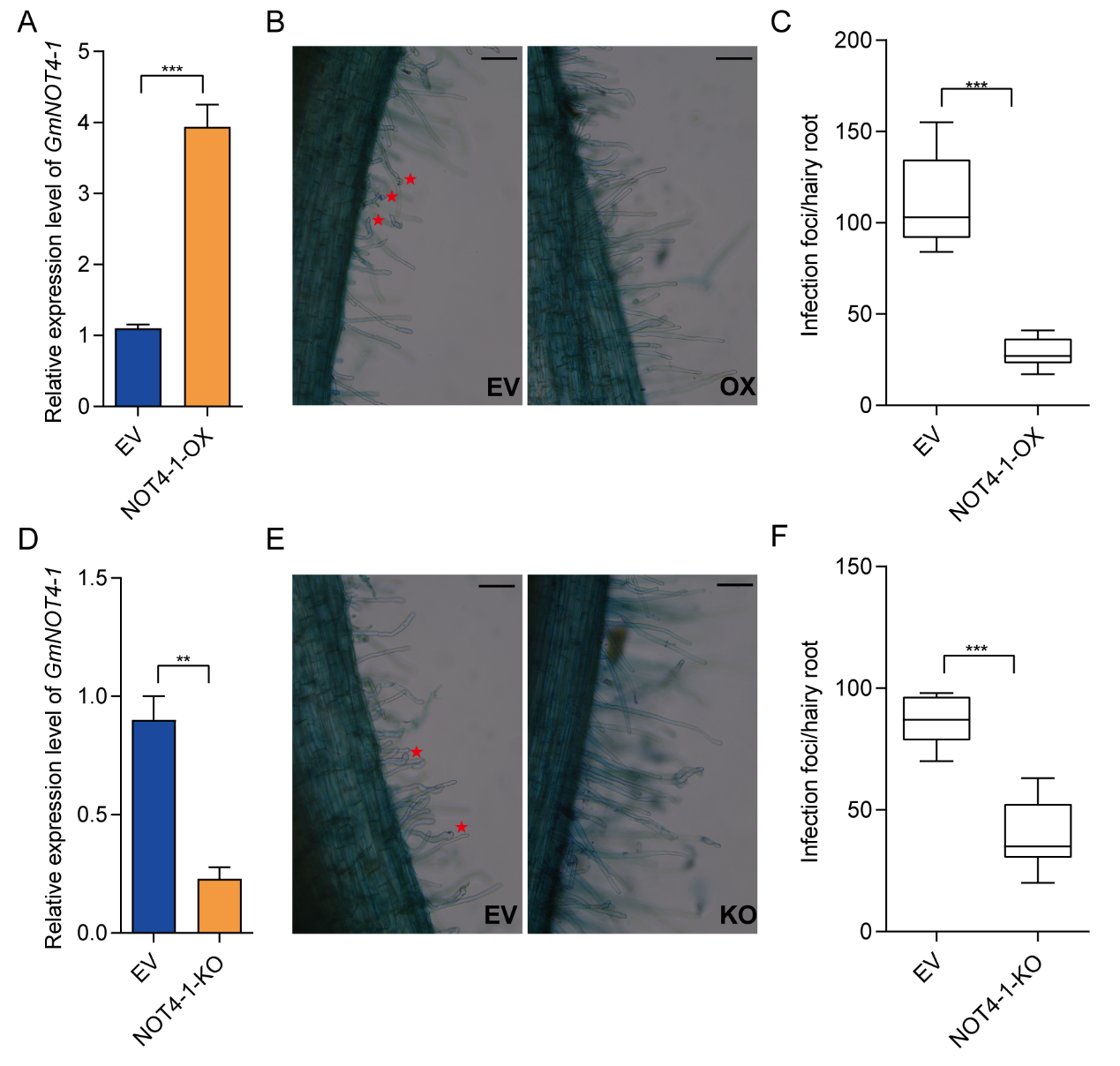

Supplement: Supplementary Figure 6 — GmNOT4-1 overexpression and knock out decreases the number of deformed root hairs (A), Expression level of transgenic hairy roots harboring empty vector and 35S:GmNOT4-1. The expression levels were normalized against the housekeeping gene of soybean GmCYP2. Student’s t-test was performed (***p < 0.001, n = 20). (B), At 6 DAI, 2 cm root segments of hairy roots overexpressing GmNOT4-1 or expressing EV below the root-hypocotyl junction were cut and stained with 1% (w/v) methylene blue. Deformed root hairs were counted (n=20). (B) Root hair deformation in transgenic roots harboring EV and 35S:GmNOT4-1 vector. Bar=40 μm. (C), Quantification of deformed root hairs in the transgenic lines (n=10 to 12). Values are averages ± SD from three independent experiments. Asterisks represent statistically significant differences. (n = 20, Student’s t-test; ***p < 0.001). (D), Expression level of transgenic hairy roots harboring empty vector and GmNOT4-1-KO. The expression levels were normalized against the housekeeping gene of soybean GmCYP2. Student’s t-test was performed (***p < 0.001, n = 20). (E), Root hair deformation in transgenic roots harboring EV and GmNOT4-1-KO. Bar=40 μm. (F), Quantification of deformed root hairs in the transgenic root harboring EV and GmNOT4-1-KO (n=20). Values are averages ± SD from three independent experiments. Asterisks represent statistically significant differences. (n = 20, Student’s t-test; ***p < 0.001). [file Image_6.tif]

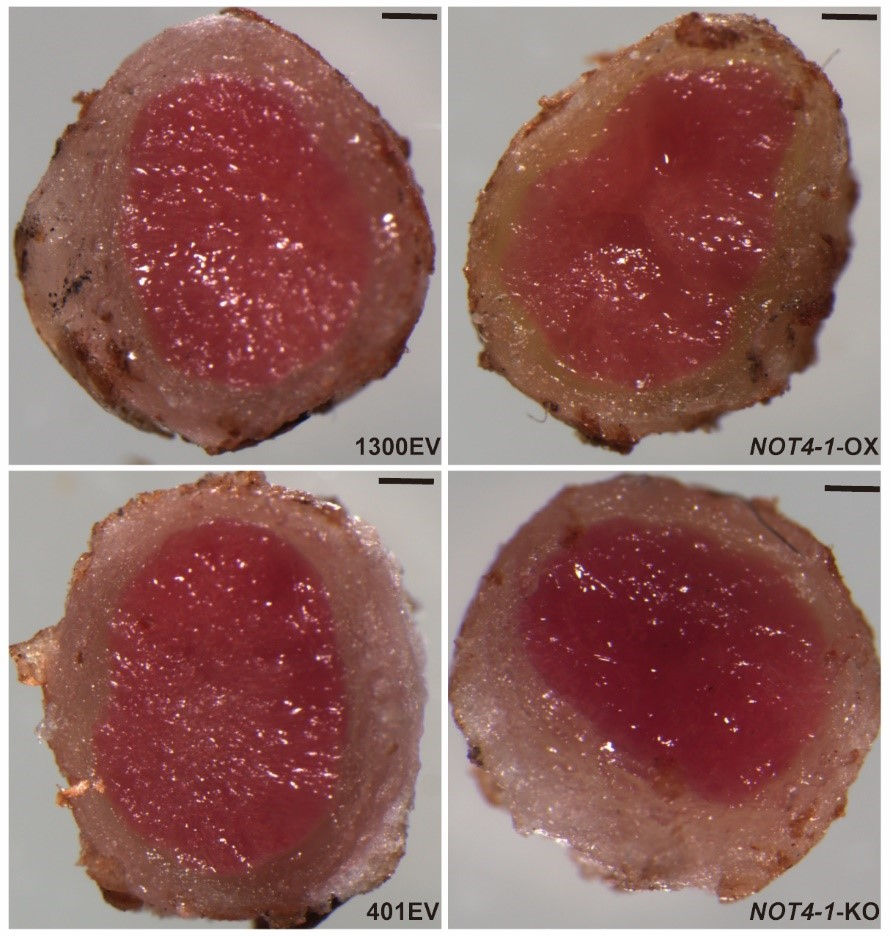

Supplement: Supplementary Figure 7 — Nodules of GmNOT4-1 overexpression and RNAi roots are functional Nodule performance of mature nodule in roots expressing empty vector, overexpression, and GmNOT4-1-KO at 28 DAI. Bar =2 mm. [file Image_7.tif]

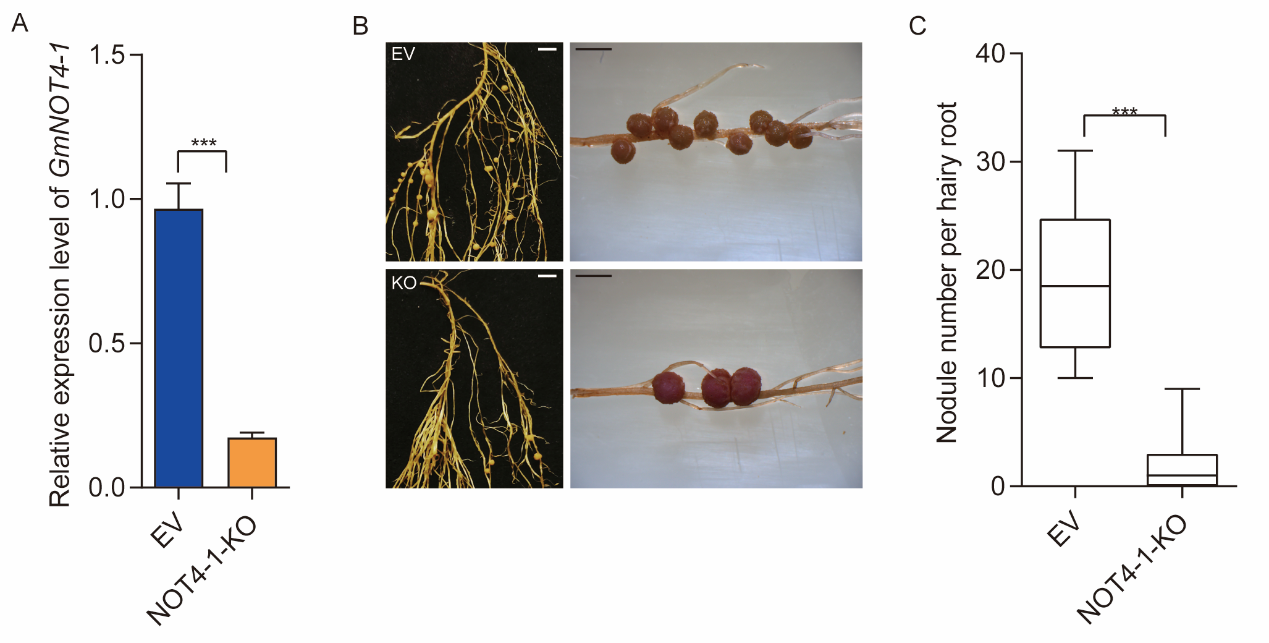

Supplement: Supplementary Figure 8 — Phenotypic analysis of GmNOT4-1 gene editing (A), Relative expression level of GmNOT4-1 in GmNOT4-1 edited roots; The expression levels were normalized against the housekeeping gene of soybean GmCYP2. Student’s t-test was performed (***p < 0.001, n = 15). (B), Nodule performance of individual transgenic roots expressing empty vector and GmNOT4-1-KO at 28 DAI. Bar =2 mm. (C), Quantitative data of nodule number per hairy root carrying empty vector and GmNOT4-1-KO at 28 DAI. Values are the mean ± SD. 20 hairy roots were collected for each biological replicate. (Student’s t-test; ***p < 0.001). [file Image_8.tif]

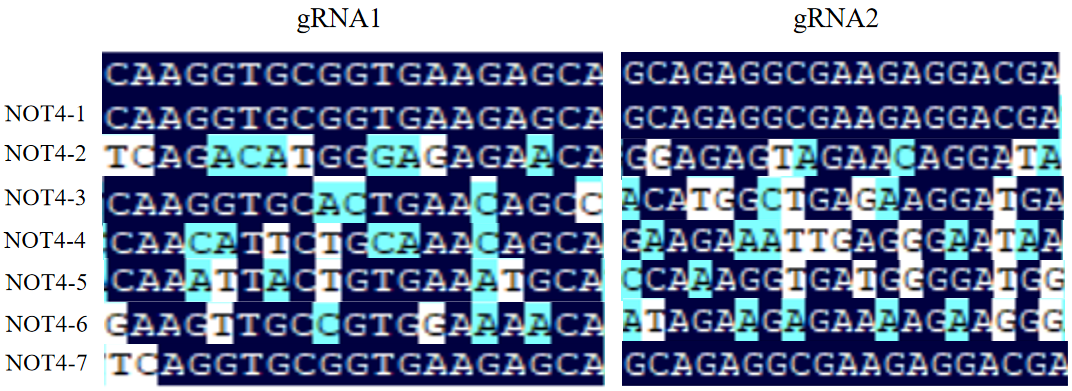

Supplement: Supplementary Figure 9 — Alignment of GmNOT4-1 gRNAs with other GmNOT4 family members (A), The front gRNA sequence aligned with the corresponding region of other GmNOT4 family members. (B), The back gRNA sequence aligned with the corresponding region of other GmNOT4 family members, the software Daneman was applied for the sequence alignment. [file Image_9.tif]
